# A Promising Anticancer Agent Dimethoxycurcumin: Aspects of Pharmacokinetics, Efficacy, Mechanism, and Nanoformulation for Drug Delivery

**DOI:** 10.3389/fphar.2021.665387

**Published:** 2021-07-06

**Authors:** Muhammad Sohail, Wenna Guo, Xin Yang, Zhiyong Li, Yanli Li, Hui Xu, Feng Zhao

**Affiliations:** ^1^ School of Pharmacy, Collaborative Innovation Center of Advanced Drug Delivery System and Biotech Drugs in Universities of Shandong, Key Laboratory of Molecular Pharmacology and Drug Evaluation (Yantai University) Ministry of Education, Yantai University, Yantai, China; ^2^ School of Chemistry and Chemical Engineering, Yantai University, Yantai, China; ^3^ Department of Pharmaceutics, Binzhou Hospital of TCM, Binzhou, China

**Keywords:** anticancer agent, dimethoxycurcumin, pharmacokinetics, mechanism of action, nanoformulation

## Abstract

Curcumin is a well-known anticancer natural product with various significant bioactivities that has been well documented, but its widespread use is mainly hindered by insufficient ADME properties such as poor solubility and low metabolic stability. Dimethoxycurcumin (DiMC) is a kind of lipophilic compound derived from curcumin that maintains its anticancer potency and has greatly improved systematic bioavailability. Therefore, DiMC is regarded as a promising plant-derived anticancer agent that deserves to be well developed. Herein, we concentrate on the published work by those from original research groups concerned with the pharmacokinetics, efficacy, and mechanism of DiMC involved in the treatment of various tumors, as well as the nanoformulations for effective drug delivery.

## Introduction

Curcumin is the principal curcuminoid of turmeric (*Curcuma longa* L.), a member of the ginger family. As a kind of natural product, curcumin has been extensively noticed in herb medicine due to various striking bioactivities beneficial to human health such as antioxidant, antiviral, anti-inflammatory, and antiangiogenic effects, especially the great potential of the anticancer effect by disturbing multiple molecular targets (Yang et al., 2017). However, the pharmaceutical function and clinical application of curcumin are severely limited by low water solubility, instability, poor absorption, and rapid systemic elimination *in vivo* (Sun et al., 2018). Therefore, a great deal of research has long been performed to find appropriate solutions to the problems mentioned above, for which chemical structure modification provides an effective way and attracts much interest (Yallapu et al., 2013; Heger et al., 2014).

**FIGURE 1:**
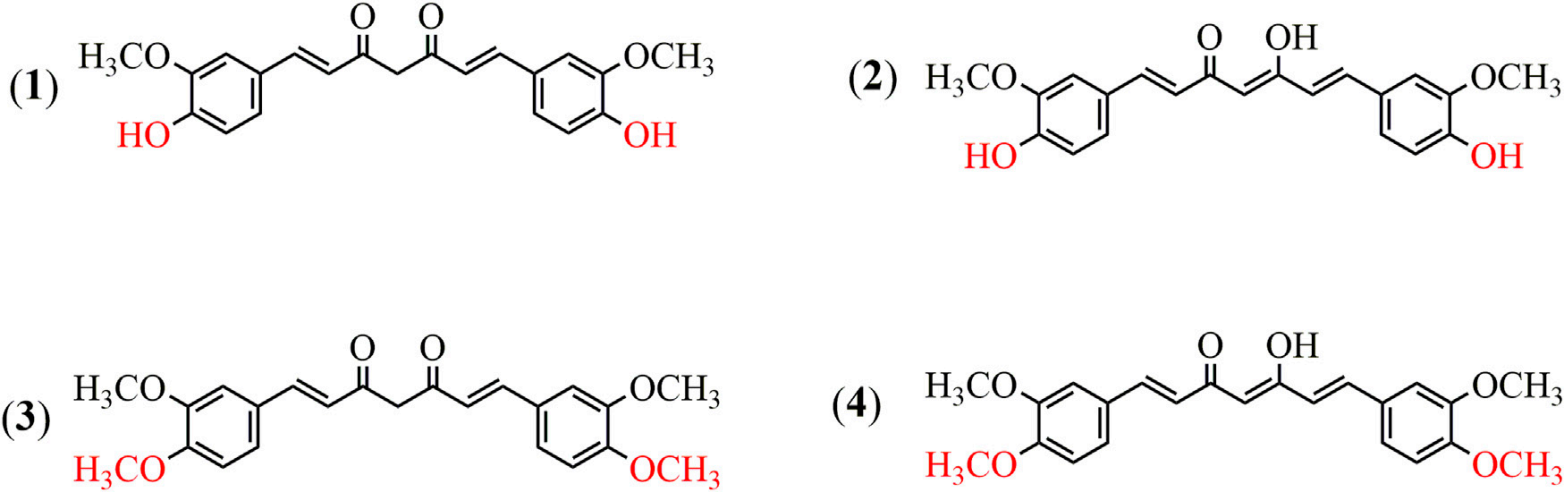
Chemical structures of curcumin and dimethoxycurcumin. **(1)** Curcumin (the bis-keto form). **(2)** Curcumin (the enolate form). **(3)** Dimethoxycurcumin (the bis-keto form). **(4)** Dimethoxycurcumin (the enolate form).

Dimethoxycurcumin (DiMC), also called dimethylcurcumin or ASC-J9, is a lipophilic compound structurally derived from curcumin with a minor modification of both hydroxyls into methoxyls (Lin et al., 2013; Liu et al., 2015). As illustrated in Figure 1, DiMC is chemically similar to curcumin, and they both have tautomerism between the bis-keto and enolate forms depending on the pH value of the medium, while the methylene group among the β-diketone structure provides them with similarly remarkable antioxidant properties (Hadjidemetriou et al., 2013). Meanwhile, the methylation of both hydroxyl groups makes DiMC more stable and lipophilic than curcumin, further providing it with a significantly reduced degradation rate and a much improved drug delivery system (Hassan et al., 2015; Dützmann et al., 2016; Jayakumar et al., 2016). It was also found that DiMC could respond to normal healthy cells in a way that is similar to curcumin but exerted more potent antioxidant properties (Kunwar et al., 2011a; Kim et al., 2016; Arrue et al., 2017). Resultantly, DiMC usually displays various pharmacological activities that are mostly maintained and even more improved in contrast to curcumin, which mainly includes anti-inflammatory, antihypertensive, neuroprotective, nephroprotective, and anticancer effects that are primarily attributed to its stability and potent anti-oxidant and free radical scavenger properties (Karimian et al., 2017). Much attention has lately been given to DiMC due to its potency against various cancer cell lines in many cases, such as the ability to serve as an androgen receptor antagonist in human prostate cancer cells *in vivo* (Ramkumar et al., 2017; Hatamipour et al., 2018; Hatamipour et al., 2019). Recent research has further revealed the miraculous power of DiMC as an anticancer agent *via* special actions on various molecular targets, including inactivation of enzymes, regulation of P-glycoprotein activity, inducing apoptosis and promoting autophagy of cancer cells, inhibiting migration, and DNA transcription (Munigunti et al., 2014; Yoon et al., 2014).

In order to provide relatively comprehensive basic data for further development, the present review systematically summarized the literature about the promising anticancer agent DiMC for the first time, which mainly included its pharmacokinetic characteristics, anticancer effects against various cancer cells, and the molecular mechanism of action. The research on drug delivery systems was also involved, especially the nanoformulations of DiMC that focused on improving stability and ADME profiles *in vivo*.

## Pharmacokinetics of DiMC

As previously described, DiMC is more effective in inhibiting proliferation and inducing apoptosis of different types of human cancer cells than curcumin and its analogs. In addition, in terms of pharmacokinetics, DiMC also has multiple advantages compared with curcumin. It has been reported that the reason behind the low bioavailability of curcumin was its rapid metabolization into tetrahydrocurcumin and some other less active product *in vivo* (Wang et al., 2019). DiMC is a methylated analog of curcumin, where the phenolic -OH groups in curcumin are replaced with methoxyl groups and the symmetric structure offers better chemical and biological stability.

Hassan et al. found that DiMC could effectively induce cell apoptosis and cell cycle arrest even at low concentrations, which might be due to metabolic stability. For further verification, the authors compared the metabolism of curcumin and DiMC *in vivo* by using human liver microsomal enzymes at different time intervals. The results showed that the retention time of curcumin and DiMC was 2.3 and 4.8 min, respectively, and the inherent clearance rate conformed to the first-order kinetics. In the meantime, it was found that in the presence of UDPGA (reflecting phase II metabolism), DiMC was more stable than curcumin, while in the absence of UDPGA, DiMC was less stable than curcumin (Hassan et al., 2015). It is speculated that because hydroxyl group compounds are easily exposed to phase II metabolism (glucuronidation), DiMC is more resistant to phase II metabolism than curcumin under physiological conditions and at the same molar concentration.

In addition, consistent research studies of the modern era have demonstrated that even at higher doses, the bioavailability and metabolic stability of DiMC were higher than those of curcumin. At high doses (10–20 µM), the clearance rate of DiMC was significantly lower than that of curcumin (*p* < 0.05), and the plasma stability of DiMC was 3 times that of curcumin at a dose of 5 mg/kg (Tamvakopoulos et al., 2007; Mach et al., 2010; Hassan et al., 2015). The higher oral bioavailability and metabolic stability of DiMC could to some extent make up for the clinical limitations of curcumin and at the same time significantly improve its ability to induce apoptosis of cancer cells (Chainoglou and Hadjipavlou, 2019). Therefore, DiMC may be the better choice for effective treatment of cancer with lower side effects (Zhang et al., 2017).

## Antitumor Effects and Mechanisms

**FIGURE 2:**
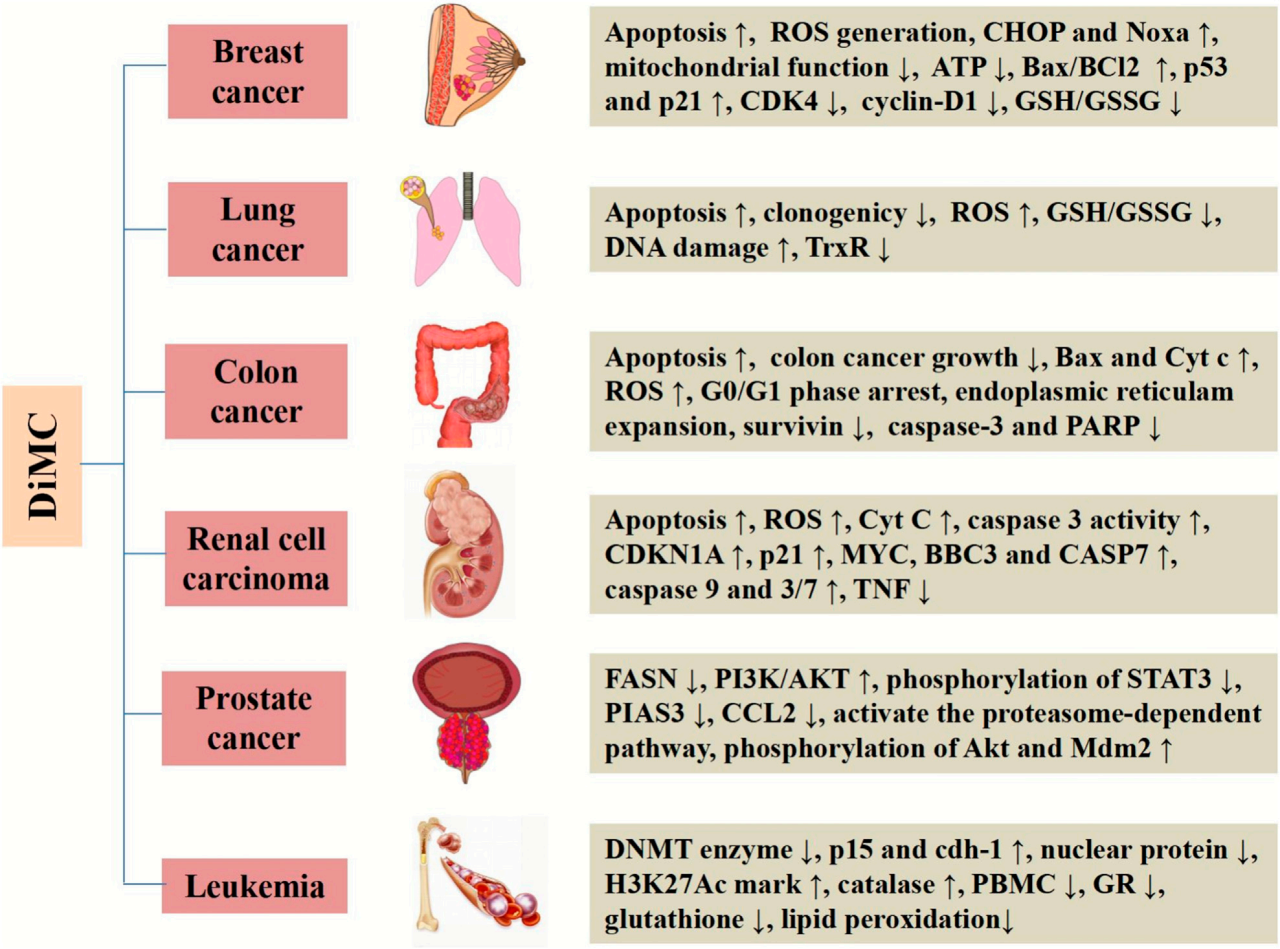
Systemic activities and mechanisms of DiMC against various cancers.

DiMC has good metabolic stability and a wide range of pharmacological activities. It is provided with significantly anti-inflammatory, antihypertensive, neuroprotective, anti-infection, nephroprotective, and antifungal effects. More importantly, DiMC has a significant therapeutic effect on a variety of life-threatening diseases including cancer. DiMC can produce different cytotoxicities due to the difference in the uptake of tumor cells and normal cells, thereby producing significant anticancer effects while protecting normal cells, and the anticancer effect is better than that of curcumin. The difference in chemical structure is the fundamental reason for the different pharmacokinetics, epigenetic performance, transcription process, and cellular uptake of DiMC. In this section, we will briefly highlight DiMC’s anticancer activity and the mechanism of action (Table 1 and Figure 2).

**TABLE 1:** Anti‐cancer activities of DiMC on different cancer cell line models.

Model	Dose	Duration of treatment	Outcomes	Reference
MCF-7 and T47D	0–30 µM *in vitro* and 25 mg/kg *in vivo*	20 days *in vivo* and 0–24 h *in vitro*	Cellular vacuolation, ROS ↑, ubiquitinated proteins ↑, ER 37 stress-related ATF438 and CHOP 39 ↑, and Bim 40 and Noxa proteins ↑	Yoon et al. (2014)
MCF-7	5–50 µM	2–6 h	ATP/ADP ↓, DNA damage, ATP synthase subunits ↓, p53 and p21 ↑, CDK4 and cyclin-D1 ↓, S-phase cell cycle arrest, Bax/BCl2 ↑, ROS ↑, GSH/GSSG ↓, and mitochondrial membrane potential ↓	Kunwar et al. (2012)
A549	1.5–10 µM	15 min−48 h	Apoptosis ↑, clonogenicy ↓, ROS ↑, GSH/GSSG ↓, DNA damage ↑, and TrxR ↓	Jayakumar et al. (2016)
SW480 and SW620	25–30 µM	48 h *in vitro*	Apoptosis ↑, colon cancer growth ↓, protein expression of Bax and Cyt c ↑, ROS ↑, G0/G1 phase arrest, and endoplasmic reticulum expansion	Zhao et al. (2017)
HT-29 and SW480	12.5–100 µM	24–72 h	Apoptosis ↑, survivin ↓, and caspase-3 and PARP ↓	Chen et al. (2016)
Caki cell	40–80 µM	6–24 h	Apoptosis ↑, ROS ↑, Cyt C ↑, and caspase 3 activity ↑	Lee et al. (2010)
786-O	10–40 µM	24 h	CDKN1A ↑, p21 ↑, MYC, BBC3, and CASP7 ↑, caspase 9 and 3/7 ↑, and TNF ↓	Zanetti et al. (2021)
LNCaP	5 µM	24 h	Activation of the proteasome-dependent pathway and phosphorylation of Akt and Mdm2 ↑	Lai et al. (2013)
C4-2 and LNCaP	10 µM	0–24 h	FASN ↓ and PI3K/AKT ↑	Wen et al. (2016)
PC3	5 µM	24 h	Phosphorylation of STAT3 ↓, PIAS3 ↓, and CCL2↓	Lin et al. (2013)
CEM and Jurkat	2 µM	24 h	p15 and cdh-1 ↑, DNMT enzyme ↓, nuclear protein ↓, and H3K27Ac mark ↑	Hassan et al. (2016)
PBMC	5 µM	24 h	Catalase ↑, PBMC ↓, GR ↓, glutathione ↓, and lipid peroxidation↓	Simon et al. (2018)

### Breast Cancer

Breast cancer is a common malignant tumor and a serious threat to the lives of women. The morbidity and mortality data of breast cancer are gradually increasing. Approximately 249,260 breast cancer cases with 40,890 deaths were revealed in the U.S. statistical report in 2016 (Siegel et al., 2016). Therefore, it is necessary to find new, alternative anticancer drugs.

DiMC has demonstrated that it has an inhibitory effect on breast cancer cells. Kunwar et al. evaluated the anticancer activity of DiMC against human breast cancer MCF-7 cells with curcumin as a positive control (Kunwar et al., 2011a). The results showed that both DiMC and curcumin could induce cytotoxicity, which increased with increasing treatment concentration from 5 to 50 μM. Concurrently, they both also showed similar concentration-dependent cytotoxicity to spleen lymphocytes, but the level of cytotoxicity was much lower than that of MCF-7 cells. The results suggested that DiMC has selective toxicity to breast cancer cells. Another study by Kunwar et al. found that the basal ROS level of MCF-7 cells stained with dihydroethidium increased significantly after 2 h of DiMC treatment, that is, DiMC had a pro-oxidative effect on MCF-7 cells, which was equivalent to that of curcumin (Kunwar et al., 2012). Yoon et al. further compared the anticancer activity of DiMC and curcumin on various breast cancer cells (Yoon et al., 2014). The results showed that DiMC had a more potent *in vitro* anticancer effect than curcumin on breast cancer cells T-47D, MCF-7, MDAMB435S, and MDA-MB 231. Further *in vivo* pharmacodynamics research in human breast cancer–bearing nude mice found that both DiMC and curcumin reduced tumor volume in a dose-dependent manner, but the tumor-reducing effect of DiMC at 25 mg/kg was greater than that of curcumin at 50 mg/kg. It suggested that the anticancer effect of DiMC *in vivo* was greater than that of curcumin, and a lower concentration of DiMC could produce a stronger anticancer effect.

**FIGURE 3:**
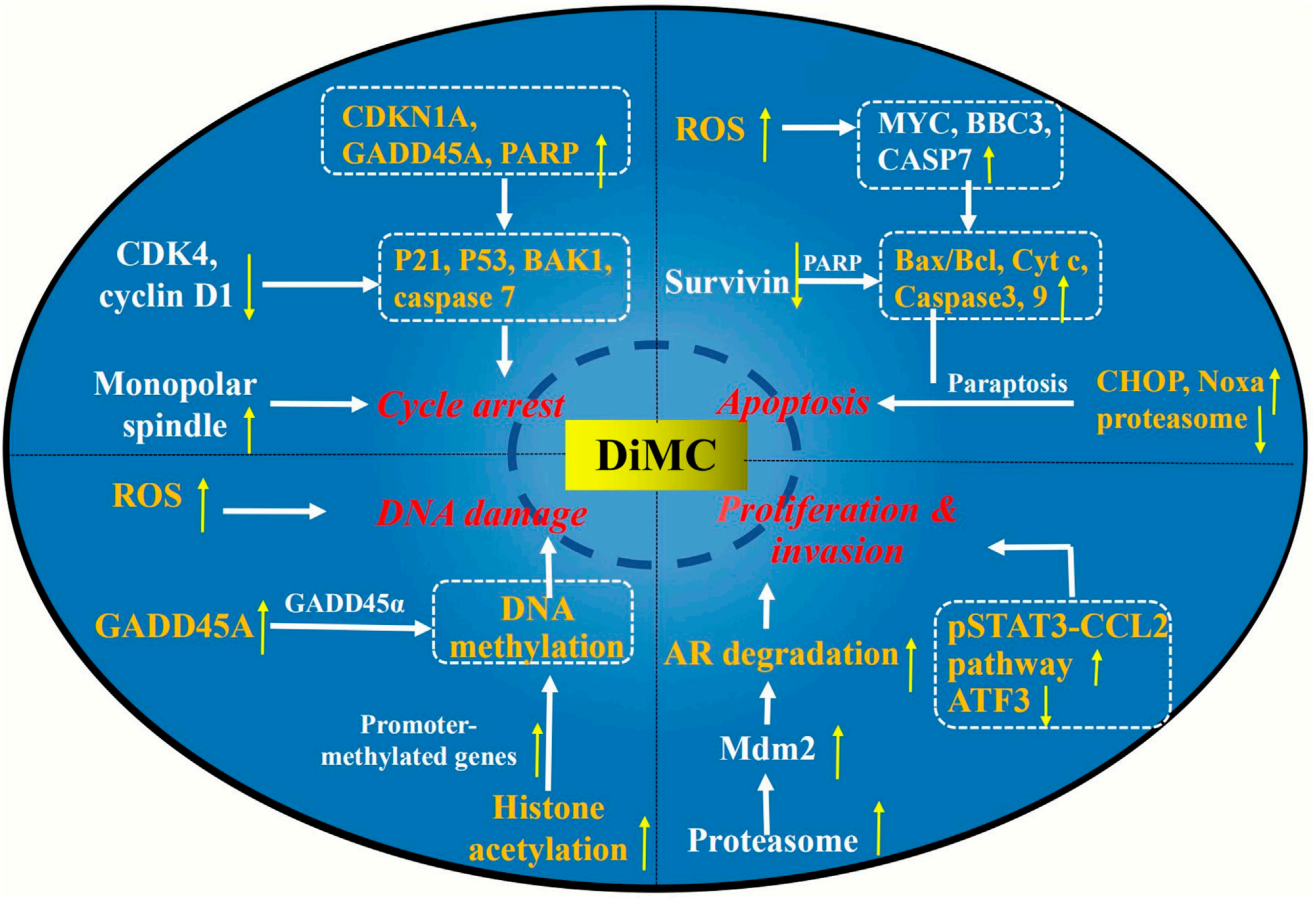
Major anticancer mechanisms of DiMC.

The elucidation of the mechanism is of great significance for disease treatment and drug discovery. Studies have found that there were complex mechanisms of DiMC against breast cancer, mainly related to oxidative stress, proteasome inhibition, mitochondrial dysfunction, and regulation of the expression of related signaling pathway factor genes and proteins (Figure 3).

Excessive reactive oxygen species (ROS) can oxidize specific chemical clusters in cells, further lead to DNA mutations, or activate the release pathway of inflammatory factors, which can eventually lead to the activation of oncogenes or the initiation of normal cell apoptosis (Moloney and Cotter, 2018). Therefore, controlling the content of ROS in tumor cells can effectively promote cell apoptosis and inhibit cell differentiation. Kunwar et al. showed that the production of intracellular ROS was decreased when the concentration of DiMC was 5 μM, while at 25 and 50 μM, the production of intracellular ROS was significantly increased (Kunwar et al., 2011b). It was speculated that the increase in the ROS level observed after DiMC treatment might be due to intracellular production or the diffusion of exogenous ROS. Furthermore, with the known cell-permeable free radical scavenger N-acetylcysteine as the positive control, the viability of DiMC (50 μM)-treated cells was evaluated. It was found that N-acetylcysteine inhibited DiMC-induced cytotoxicity with a concentration dependency, confirming the involvement of ROS in DiMC-induced toxicity (Kunwar et al., 2012). Yoon et al. also found that DiMC increased the level of ROS in MDA-MB 435S cells to a greater extent than curcumin (Yoon et al., 2014).

Paraptosis is an alternative, non-apoptotic form of programmed cell death (Fontana et al., 2020), and various stimuli, including paclitaxel, curcumin, and ophiobolin A, reportedly induce paraptosis or paraptosis-like cell death in resistant malignant cancer cells (Lee et al., 2016; Kessel, 2019). Yoon et al. found that DiMC inhibited proteasome activity more strongly than curcumin, which might lead to severe endoplasmic reticulum stress and promote the observed expansion (Yoon et al., 2014). Concurrently, DiMC treatment could upregulate the protein levels of CCAAT–enhancer-binding protein homologous protein (CHOP) and Noxa, which is a candidate mediator of p53-induced apoptosis. It was suggested that DiMC could selectively induce paraptosis of breast cancer cells while retaining normal cells. Furthermore, DiMC could also induce oxidative stress, leading to further weakening of proteasome activity (Yoon et al., 2014).

Moreover, the mechanisms of DiMC against breast cancer were also related to DNA damage, mitochondrial function, expression of cell cycle regulatory proteins, and induction of apoptosis (Kunwar et al., 2012). DiMC could induce the production of ROS, which leads to mitochondrial dysfunction and cell death. In the meantime, DiMC could also reduce the level of ATP in cells by downregulating ATP synthase subunits, resulting in a state of energy depletion. At higher concentrations (25 and 50 μM), DiMC could also induce secondary apoptosis or necrotic cell death in MCF-7 cells by regulating the expression of proapoptotic proteins Bax and Cytochrome c (Cyt c) and antiapoptotic protein Bcl-2. In addition, it has been found that DiMC could inhibit the expression of cyclin-dependent kinase four (CDK4) and cyclin-D1 while inducing the expression of p53 and p21, which may play a key role in cell cycle arrest (Kunwar et al., 2012).

### Lung Cancer

In general, the untreated nodules in the lungs are identified as a pulmonary cancer. Approximately 224,390 lung cancer cases with 158,080 deaths were revealed in the U.S. statistical report in 2016, of which the disease/death ratio was 117,920/85,920 for males and 106,470/72,160 for females, respectively (Siegel et al., 2016).

Lung adenocarcinoma is a common type of non–small-cell lung cancer. Wada et al. studied the drug resistance of 37 novel curcumin analogs including DiMC and curcumin *in vitro* using gefitinib-resistant lung adenocarcinoma cell lines CL1-5 and H1975 (Wada et al., 2015). The results showed that compared with other curcumin analogs, DiMC significantly decreased the total epidermal growth factor receptor (EGFR) and pEGFR levels of CL1-5 cells carrying wild-type EGFR and also decreased the expression level of total EGFR in EGFR double mutated gefitinib-resistant H1975 cells. The expression level of total EGFR effectively induced gefitinib-insensitive EGFR degradation. It is speculated that the existence of methoxyl groups of C3′ and C4′ in the DiMC chemical structure is beneficial to the EGFR inhibitory activity of gefitinib resistance. Jayakumar et al. investigated the radiosensitizing effect of DiMC in A549 lung cancer cells with curcumin as a positive control. At 2.5 mM, DiMC combined with radiation could significantly increase the apoptosis and mitotic death of A549 cells. In contrast, curcumin at this concentration of 2.5 mM neither showed toxicity toward tumor cells nor exhibited radiosensitizing activity (Jayakumar et al., 2016).

Further study on the possible mechanism of DiMC against lung cancer found that DiMC enhanced the radiosensitivity of lung cancer cells mainly by participating in the regulation of ROS and thioredoxin reductase (TrxR) (Figure 3). After DiMC was combined with radiation, the intracellular ROS level increased significantly, the GSH/GSSG ratio decreased significantly, and DNA repair was significantly slowed down, indicating that the treatment with DiMC could make cells sensitive to radiation-induced cytotoxicity by promoting oxidation and inhibiting DNA repair. The thioredoxin system is one of the main antioxidant systems, which can maintain redox balance and participate in DNA synthesis and apoptosis, and is related to radiation resistance of tumor cells. Jayakumar et al. found through computer docking analysis that the binding energies of DiMC and curcumin with TrxR were 7.4 and 7.6 kcal/mol, respectively, which showed high affinity binding and indicated that they could be potential inhibitors of TrxR. The cell experiment further found that DiMC inhibited TrxR in a dose-dependent manner with a half maximal inhibitory concentration (IC_50_) value of 5.4 mM. It was inferred that DiMC could increase oxidative stress by inhibiting TrxR, thus slowing down DNA repair and inhibiting DNA synthesis, and resulting in mitotic death (Jayakumar et al., 2016).

### Colon Cancer

Colon cancer is the third most prevalent malignant tumor in the world, with an incidence of 10.2% and a mortality rate of 9.2% of all cancer types. It has a trend of increasing year by year, seriously threatening human health. DiMC is a potential candidate for the treatment of colon cancer. It has increased potential to induce apoptosis in colon cancer cells with less toxicity to normal cells and has a higher bioactivity than curcumin.

Chen et al. found that the IC_50_ for DiMC was 43.4 and 28.2 μM on colon cancer cells HT-29 and SW480 (Chen et al., 2016). Simultaneously, the tumors of SW480 and HT29 cells treated with DiMC were significantly smaller than those in mice treated with normal saline. It was suggested that DiMC significantly inhibited the growth of colon cancer cells in a dose-dependent manner. In contrast, the IC_50_ for DiMC was 454.8 μM on normal colonic mucosal epithelial cell NCM460, which was much higher than that of colon cancer cell lines, indicating that DiMC was less toxic to normal colonic epithelium. Flow cytometry further showed that compared with curcumin, the inhibition rate of proliferation of DiMC was higher, the rate of apoptosis increased, and the cell density decreased at a concentration of 5–15 μM (Tamvakopoulos et al., 2007). It is speculated that the phenolic -OH groups in curcumin are replaced with methoxyl groups, and the symmetric structure in DiMC offered better chemical and biological stability.

5-fluorouracil (5-Fu) is the standard chemotherapy treatment for colon cancer. However, the response rates are only 10–15% as a result of the severe side effects and resistance. Zhao et al. investigated the anticancer efficacy of DiMC and 5-fluorouracil (5-FU) in colon cancer cell lines SW480 and SW620 (Zhao et al., 2017). The results showed that both DiMC and 5-Fu significantly inhibited the growth of SW480 and SW620 cells in a dose-dependent manner, and the maximum effect was observed at 128 μmol/L and 128 mg/L, respectively. Moreover, they both had an additive anticancer effect on colon cancer cells. From the information above, DiMC can significantly inhibit the proliferation of colon cancer cells in a dose-dependent manner *in vitro* and *in vivo*.

As a potential therapeutic agent for colon cancer, the mechanism of DiMC has also been deeply studied (Figure 3). Caspase-3 is the most important effect or protease in apoptosis, which can be activated by enzymatic cleavage to regulate the apoptotic cascade (Zhou et al., 2018). Survivin (BIRC5) belongs to the apoptotic (IAP) gene family and is thought to be able to directly inhibit the activity of caspase-3 to prevent apoptosis in various cancer tissues and cancer cell lines (Jaiswal et al., 2015; Li et al., 2019). Chen et al. found that DiMC treatment could downregulate survivin, while caspase-3 and PARP (the specific substrate of caspase-3) were cleaved to its active fragments (Chen et al., 2016). The results suggested that DiMC might activate caspase-3 by downregulating survivin to induce apoptosis. In addition, the mobility of DiMC-treated cells decreased significantly, which might be related to the upregulation of E-cadherin expression (Chen et al., 2016).

Many biological processes such as oxidative stress, cell apoptosis, cell cycle phase arrest, and mitochondrial membrane potential play key roles in tumor regulation. Zhao et al. found that DiMC could also increase the level of ROS to upregulate CHOP and Noxa to prevent and treat colon cancer (Zhao et al., 2017). At the same time, DiMC could also induce apoptosis of colon cancer cells by increasing the expression of Bax and Cyt c and reducing the expression of Bcl2. In addition, the anti–colon cancer activity was also closely related to the induction of G0/G1 phase arrest, endoplasmic reticulum expansion, and decreased mitochondrial membrane potential (Zhao et al., 2017).

### Renal Cell Carcinoma

Renal cell carcinoma (RCC) is one of the common clinical malignant tumors of the genitourinary system, accounting for 90% of the primary malignant tumors of the kidney in adults. At present, curcumin has been widely proven to have a clear therapeutic effect on RCC. Curcumin can exert its anti-RCC activity by inhibiting the NF-κB signal pathway, regulating autophagy, regulating the RK5/AP-1 pathway, and so on (Li et al., 2017a; Deng et al., 2018; Zhang et al., 2020). DiMC, as a curcumin analog, has higher biological stability and lower metabolism *in vivo*, so it has been paid attention to with regard to the treatment of RCC. Several studies have also demonstrated that DiMC also has an effect of anti-RCC which is higher than that of curcumin.

Lee et al. compared the effect of DiMC and bis-demethoxycurcumin to induce apoptosis in human RCC Caki with curcumin as a positive control (Lee et al., 2010). The results demonstrated that at 80 μM, the three compounds significantly reduced the cell viability, and DiMC was the most potent compound, followed by curcumin and bis-demethoxycurcumin. The typical ladder pattern of DNA fragmentation, which is considered a hallmark of apoptotic cell death, was observed when Caki cells were treated for 24 h. It was suggested that curcumin, DiMC, and bis-demethoxycurcumin could reduce cell viability by inducing apoptosis, and DiMC has the strongest anti–renal cell carcinoma activity. It was speculated that the methoxyl groups contribute to the enhancement of cell apoptosis. The latest study showed that DiMC also has better anti-RCC activity than curcumin and demethoxycurcumin in human RCC line 786-O (Zanetti et al., 2021). In this study, curcumin, demethoxycurcumin, and DiMC exerted their cytotoxic effects in a dose-dependent manner, and DiMC had the most significant antitumor activity. In addition, demethoxycurcumin and DiMC also showed genotoxicity (Zanetti et al., 2021).

The mechanism of DiMC inhibiting the activity of RCC is mainly related to oxidative stress, DNA damage, cell cycle arrest, and induction of cell apoptosis (Figure 3). Excess ROS can easily lead to the depolarization of the mitochondrial membrane and releases proapoptotic molecules from mitochondria into the cytosol, which may act to induce apoptosis. Moreover, the release of Cyt c from the mitochondrial membrane leads to an increased level of Cyt c in the cytoplasm and nucleus, which may activate caspase-9 and trigger the effector caspase-3, to eventually lead to apoptosis. DiMC could induce apoptosis by increasing the level of ROS in Caki cells, promoting the release of Cyt c and the activation of caspase-3 (Lee et al., 2010). DNA damage is one of the main ways to inhibit the proliferation of tumor cells. RCC treated with DiMC could increase the expression of the GADD45A gene, which encodes the GADD45 *α* protein, and participated in DNA repair and induced DNA damage. The cell cycle is the basic process of cell life activities, and cyclin-dependent kinase (CDK) is one of the main related molecules in cell cycle regulation. CDKN1A encodes for the p21 protein involved in cell cycle regulation and is an important inhibitor of CDK. In the 786-O RCC line, DiMC could activate the p21 protein by upregulating the expression of CDKN1A and then induce cell cycle arrest (Zanetti et al., 2021). In addition, DiMC could also induce apoptosis by increasing the expression of apoptosis genes MYC, BBC3, and CASP7, as well as decreasing the expression of the pro-inflammatory gene TNF (Zanetti et al., 2021).

### Prostate Cancer

The androgen receptor (AR) signaling pathway plays an important role in the development of prostate cancer. The change and reactivation of the AR signal transduction pathway are the core factors that promote the development of castration-resistant prostate cancer (CRPC) and the generation of drug resistance. ASC9, the enol isomer of DiMC, is a recently developed anti-AR agent which effectively suppresses castration-resistant prostate cancer cell proliferation and invasion.

Cheng et al. found that when dissolved in FDA-approved solvents such as DMSO, PEG-400, and Tween-80, ASC-J9 had AR degradation effects. Compared with those in AR-negative prostate cancer cells (IC_50_ 16.0 μM) or those in normal prostate cells (IC_50_ 27.0 μM), ASC-J9 was found to exhibit a decreased IC_50_ value in AR-positive prostate cancer cells (IC_50_ 6.5 μM) (Cheng et al., 2018). In another study, ASC-J9 also suppressed the tumor growth of prostate cancer cell lines C81, C4-2, and CWR22Rv1 xenografted into castrated nude mice (Lai et al., 2013). It could selectively degrade the AR protein *via* interruption of the AR–AR selective coregulator interaction. In addition, it was reported that androgens were able to induce fatty acid synthase (FASN) expression in prostate cancer, and addition of the anti-androgen might suppress the androgen-induced FASN expression. Wen et al. found that anti-androgen bicalutamide (Casodex) or enzalutamide MDV3100 had little inhibitory effect on FASN expression and FASN-mediated cell growth and invasion in prostate cancer cell lines C4-2 and LNCaP when the androgen concentration was 1 nM (Wen et al., 2016). In contrast, ASC-J9 inhibited the expression of FASN and the growth and invasion of various prostate cancer cell lines mediated by FASN.

Another study showed that ASC-J9 could not only degrade wild-type AR but also has the ability to target the AR mutant AR-F876L. The production of AR mutants will lead to the occurrence of multidrug resistance, so the consequence of suppressing AR-F876L may then abrogate AR-F876L which mediated the proliferation and metastasis of CRPC, thereby better suppressing CRPC that has already developed resistance (Wang et al., 2016). The above results suggest that ASC-J9 successfully induces the regress of the prostate cancer, which provides a new and more effective treatment against prostate cancer in the future.

The mechanisms of ASC-J9 against prostate cancer have been deeply studied, which can be divided into the AR-dependent pathway and the AR-independent pathway (Figure 3). First of all, ASC-J9 could activate the proteasome-dependent pathway to promote AR degradation through the enhanced association of the AR-murine double minute protein 2 (Mdm2) complex (Lai et al., 2013). ASC-J9 could also inhibit the proliferation and metastasis of prostate cancer by regulating pSTAT3-C-C motif chemokine-2 (CCL2) signal transduction through an AR-dependent pathway *via* inhibiting the expression of the protein inhibitor of STAT3 (PIAS3) (Lin et al., 2013). STATs are transcription factors that play an important role in the process of inflammation, and STAT3 is highly expressed and may be related to the progression of many types of cancer, including prostate cancer (Pencik et al., 2016). CCL2 is highly expressed in malignant tumor cells and may play an important role in the recruitment of tumor-associated macrophages. The regulation of the pSTAT3-CCL2 signaling pathway can better battle the growth and metastasis of prostate cancer at the castration-resistant stage (Wu et al., 2017; Lee et al., 2018). Additionally, Wen et al. also found that in AR-positive C4-2 and LNCaP cells, ASC-J9 could suppress significant FASN expression and FASN-mediated prostate cancer progression through the AR-dependent pathway (Wen et al., 2016).

In addition to the AR-dependent pathway above, the AR-independent pathway is also an important mechanism for ASC-J9 to inhibit the proliferation of prostate cancer. It was found that ASC-J9 treatment resulted in significant suppression of the STAT3 phosphorylation/activation and CCL2 expression, with little influence on the PIAS3 expression in the AR-negative PC3 cells, confirming that ASC-J9 could also go through an AR-independent pathway to modulate STAT3 phosphorylation/activation and CCL2 expression (Lin et al., 2013). Lin et al. further found that ASC-J9 could suppress prostate cancer cell invasion by inducing the sumoylation of STAT3, thereby inhibiting the STAT3 phosphorylation that led to the suppression of the EMT-SNAIL2 signals in both prostate cancer DU145 and PC3 AR-negative cells (Lin et al., 2018). Transcription factor ATF3 plays an important role in tumor formation, invasion, and metastasis by finely regulating the delicate balance between proliferation and apoptosis. ASC-J9 could increase the expression of ATF3 by reducing the expression of the glutamate–cysteine ligase catalytic subunit, thus suppressing the proliferation and invasion of prostate cancer (Tian et al., 2021).

### Leukemia

Leukemia is a malignant proliferative disease originating from hematopoietic stem cells, and its morbidity and mortality rank first among cancers in children and adolescents. At present, chemotherapy is still the main treatment method for leukemia, but the emergence of multidrug resistance often leads to an effect of chemotherapy that does not meet expectations. Finding new antitumor drugs is of great significance for the treatment of leukemia.

Epigenetic regulation is a modification mechanism that affects gene expression without DNA sequence changes, which is closely related to the occurrence and development of leukemia. DNA methyltransferase (DNMT) is an important protease related to epigenetic modification, which undertakes the *de novo* methylation of DNA. Inhibition of DNMT can induce DNA hypomethylation and restore the high expression/activation of tumor suppressor genes. Using the DNMT inhibitor 5-azacytidine (5 AC) as a positive control, it was found that at clinically relevant concentrations (2 μM), DiMC could induce the gene expression of promoter methylated genes such as p15 and CDH-1 in leukemia cells, suggesting a possible DNA hypomethylating effect by DiMC, similar to 5 AC (Hassan et al., 2015). However, DiMC did not induce the expression of any of the DNMT isotypes in leukemia cells, that is, no DNA hypomethylation activity. The above results suggested that DiMC induced the expression of promoter methylation genes through a mechanism that did not involve the reversal of DNA methylation, thereby playing a key role in the treatment of leukemia (Hassan et al., 2015).

On the basis of the studies above, Hassan et al. further investigated the cytotoxic effects and epigenetic changes associated with the combination of DiMC and the DNMT inhibitor decitabine (DAC) in primary leukemia samples and cell lines CEM and Jurkat (Hassan et al., 2016). The results clearly showed that compared with a single agent, the combination of DiMC and DAC demonstrated antagonistic cytotoxic effects and induced minimal apoptosis in primary leukemia cells. At the same time, the combination of drugs inhibited the gene expression of the DNMT enzyme and downregulated the level of nuclear protein. However, compared to the use of DAC as a single agent, the DNA hypomethylating activity of the combination did not significantly increase. In addition, the analysis of the histone marks associated with actively transcribed genes such as acetylated H3K27 (H3K27Ac) and trimethylated H3K36 (H3K36me3) near the promoter region of both genes demonstrated a significant increase in the H3K27Ac mark (Hassan et al., 2016). The above results suggest that the combination of DiMC and DAC can enhance the induction of promoter-methylated genes through the mechanism involved in increasing histone acetylation, so as to play a role in the treatment of leukemia.

Oxidative stress is closely related to the occurrence and development of leukemia. Under physiological conditions, the synergistic effect of antioxidant enzymes and antioxidants keeps free radicals at a very low level. When its production exceeds the scavenging and repairing capacity of the body, high concentrations of ROS in plasma can directly oxidize, attack, and destroy antioxidant enzymes, resulting in the decrease or loss of enzyme activity. At the same time, ROS attack the polyunsaturated fatty acids in the biofilm, triggering lipid peroxidation. Studies have shown that both curcumin and DiMC could inhibit the lipid peroxidation state of human peripheral blood mononuclear cells (PBMCs) by increasing the activity of catalase and significantly reduce the activity of glutathione reductase (GR) and the content of glutathione (Simon et al., 2018). It was speculated that DiMC may enhance the activity of catalase by increasing the level of mRNA and protein.

### Others

In addition to the cancers mentioned above, the therapeutic effects of DiMC on hepatocellular carcinoma and bladder cancer are also being studied.

Zanetti et al. evaluated the inhibitory effect of DiMC on the HepG2/C3A human hepatocellular carcinoma cell line (Zanetti et al., 2019). The results showed that DiMC had cytotoxic effect. Treatment with 10–50 μM of DiMC for 24 h decreased the cell viability from 71 to 39% in a dose–response manner, and the IC_50_ value was 37 μM. It was further found that the cytotoxicity of DiMC was mainly related to mitotic disorder and DNA damage. DiMC could cause mitotic arrest by inducing the formation of monopolar spindles. In the meantime, it could also activate the key effector factors BCL2-homologous antagonist/killer one (BAK1) and caspase-7 in cell cycle arrest and the DNA repair pathway by increasing the expression of the CDKN1A, GADD45A, and PARP1 genes, thus resulting in genotoxicity (Zanetti et al., 2019). Another study showed that sorafenib, a standard treatment to suppress the progression of hepatocellular carcinoma, combined with ASC-J9 could synergistically suppress the progression of HCC by altering cell cycle regulation, apoptosis, and invasion. Its mechanism was related to the inhibition of the expression of pSTAT3 and its downstream genes including CCL2 and Bcl2 (Xu et al., 2017).

Bladder cancer is a common malignant tumor in the urinary system. Emerging preclinical findings have indicated that androgen-mediated androgen receptor signals have been shown to correlate with the promotion of tumor development and progression (Li et al., 2017b). Studies have shown that DiMC could prevent and treat bladder cancer by inducing AR degradation (Shang et al., 2015). In a study, the daily i. p. co-injection of a carcinogen [N-butyl-N-(4-hydroxybutyl)-nitrosamine or BBN] and DiMC (75 mg/kg for 24 weeks) reduced the incidence of bladder cancer by 4-fold as compared to the DiMC-deprived group (Shang et al., 2015). DiMC treatment had little effect on serum testosterone concentration, suggesting that AR was deprived after DiMC treatment. In addition, DiMC could also be used in combination with Bacillus Calmette–Guerin, a very successful adjuvant for the treatment of bladder cancer, to better suppress bladder cancer progress (Shang et al., 2015).

## Nanoformulations of DiMC

**FIGURE 4:**
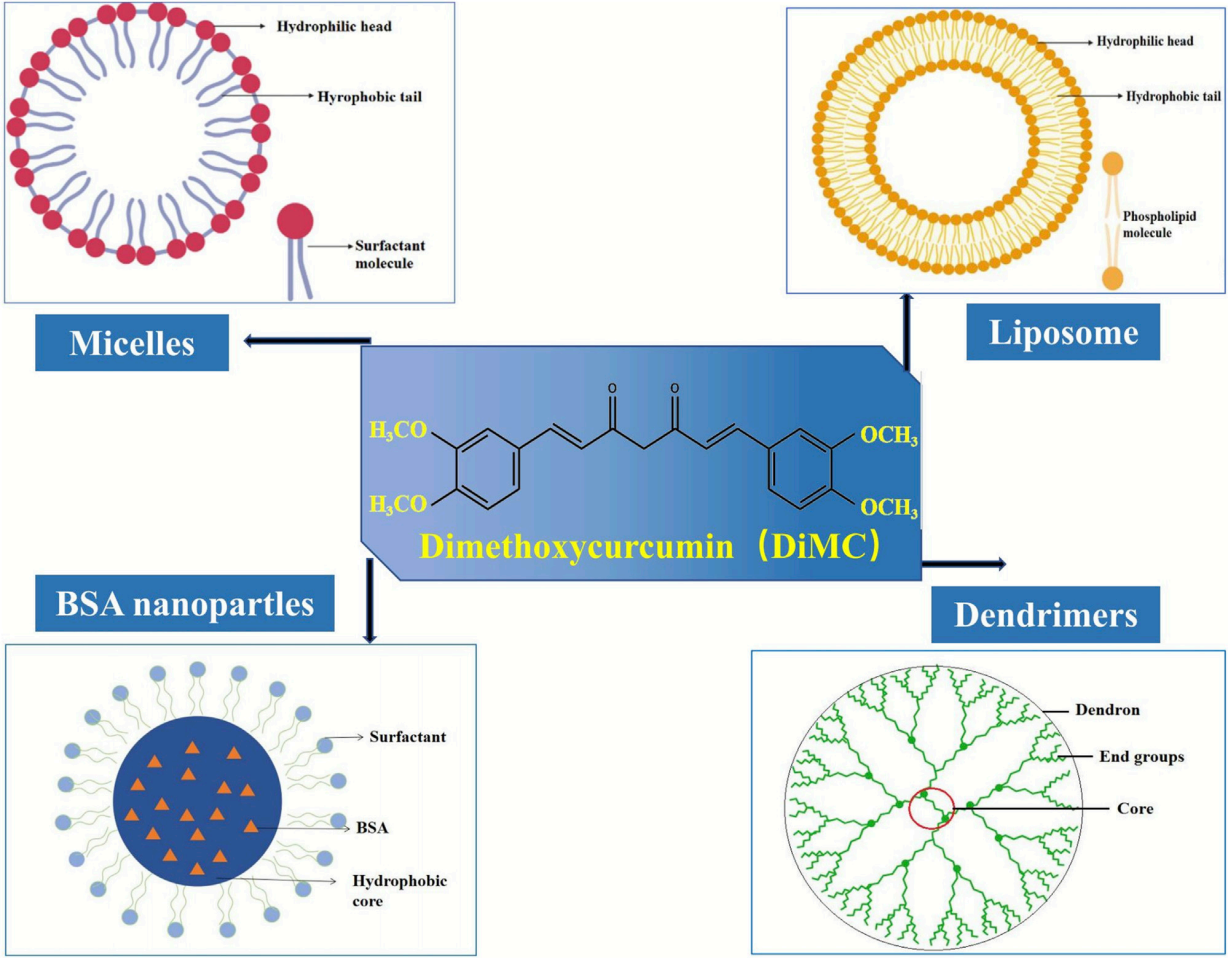
Main nanoformulations of DiMC.

The poor water solubility is one of the main challenges in cancer chemotherapy since it usually results in low bioavailability at the tumor sites and reduces therapeutic efficacy (Sun et al., 2014). It is indeed the case for DiMC, a hydrophobic analog of curcumin with superior activity against various cancer cell lines such as breast cancer, lung cancer, colon cancer, and prostate cancer. Therefore, it is a matter of the utmost importance to develop a suitable drug delivery system for further clinical application of this anticancer agent. In recent years, a certain amount of research has been carried out to deliver DiMC, mainly including some polymer-based nanoformulations (Figure 4) that provide an efficient alternative to overcome the therapeutic limitation from its poor water solubility (Table 2).

**TABLE 2:** Drug delivery systems for DiMC with improved and effective responses.

Drug delivery system	Method of preparation	Outcomes	Model	Reference
Dendrimer	Thin film	*Via* electrostatic or covalent complexation on the surface or entrapment within the dendrimer architecture; enhanced bioavailability	Spleen lymphocytes and bone marrow hematopoietic cells	Markatou et al. (2007)
BSA nanoparticles	Thermal denaturation	Mean hydrodynamic diameters ranging from 28 to 62 nm and corresponding zeta-potential values of −7.0 to −6.0 mV; increased cellular uptake and toxicity of DiMC	A549 human lung cells	Das et al. (2017); Das et al. (2019)
Liposomes	Thin-film hydration	Cholesterol played a key role in the interaction between DiMC and lipid bilayers; the DPPC:DODAP:chol:DiMC liposomal formulation (9:1:1:1 molar ratio) was the most effective with the incorporation efficiency up to 3.84%	—	Hadjideme-triou et al. (2013)
Solid dispersions	Solvent evaporation	Significantly improved drug dissolution rate; the cumulative dissolution was more than 83% in 5 min	—	Xu et al. (2019)
Polymeric micelles	Thin-film hydration	A low initial burst release followed by a sustained release *in vitro*; significantly increased drug exposure and prolonged it *in vivo*; improved the biodistribution profile of DiMC and increased drug accumulation in tumors	A549 human lung cells	Liu et al. (2015)
Stiffness-tunable nanocarriers	Thin-film hydration	Improved the cellular uptake and anticancer activity of ASC-J9; enhanced the tumor penetration in HCT 116 3D colorectal cancer spheroids	HCT 116 human colorectal adenocarcinoma cells	Tomeh et al. (2021)

### Nanoparticles

The first work reported on nanoformulation of DiMC was demonstrated by using an alternative technology of dendrimers due to the attractive properties such as well-defined structures and low polydispersity (Markatou et al., 2007). Appropriate quantities of DiMC and the water-soluble poly (amidoamine) (PAMAM) (G3.5 or G4) were dissolved in methanol and then stirred for 24 h. After evaporating under vacuum to remove the methanol completely, buffer solution was added, stirred for another 24 h in the dark, and then centrifuged to obtain the DiMC-PAPMAM complex dispersed in the aqueous medium. The results indicated that hydrophobic DiMC could be incorporated to PAMAM dendrimers in an enolic form *via* electrostatic or covalent complexation on the surface or entrapment within the dendrimer architecture, while its interaction with the integer generation dendrimer involved the major conformational change of the terminal ethylene amine groups. Although the drug–dendrimer interactions determine the bioavailability enhancement, this formulation of DiMC suffered from glaring shortcomings such as a time-consuming and complicated preparation process, as well as rather low drug-loading capacity (<5%) and poor incorporation efficiency (about 50%).

Bovine serum albumin (BSA), which has ligand-binding properties and is widely available, cheap, and easily purified, is extensively used for drug delivery and well accepted in the pharmaceutical industry. In recent years, BSA nanoparticles have been used as an effective delivery system for hydrophobic drugs like DiMC (Das et al., 2017; Das et al., 2019). Using the thermal denaturation method, BSA nanoparticles were prepared with mean hydrodynamic diameters ranging from 28 to 62 nm and corresponding zeta-potential values of −7.0 to −6.0 mV, which could easily bind DiMC to obtain drug-loaded nanoparticles. Using the A549 cell model, Das’s group further revealed such a kind of particle size effect that the cellular uptake and toxicity of DiMC increased with the increase in the particle size of these BSA-based nanoparticles (Das et al., 2017). Their latest research demonstrated significant changes in the secondary structure of BSA upon particle formation and also a decrease in helicity (Das et al., 2019). DiMC could be bound within the sub-domain IIA of BSA with a distance of about 24 Å between the hydrophobic core and the ligand, where it experienced a more rigid environment than in the native form of BSA. Also, it displayed a much higher binding constant with BSA nanoparticles than with the native BSA (1.5 ± 0.5 × 105 M^−1^ vs. 2.7 ± 0.4 × 104 M^−1^).

### Liposomes

Liposomes represent one of the most thoroughly studied categories of colloidal nanocarriers and are proven candidates for delivery of a wide range of therapeutics (Yan et al., 2020). So far, most of the research has been concentrated on developing suitable liposomal formulations of DiMC to improve the thermal stability and water solubility. Charged liposomes incorporating DiMC were investigated for the first time by using the phospholipids composed of 1,2-dipalmitoyl-sn-glycero-3-phosphocholine (DPPC), 1,2-dipalmitoyl-sn-glycero-3-phospho-(1′-rac-glycerol) (DPPG), 1,2-dioleoyl-3-dimethylammonium-propane (DODAP), and cholesterol (chol) (Hadjidemetriou et al., 2013). Three different liposomal formulations have been prepared using the thin-film hydration method, and the charged liposomes composed of DPPC:DPPG:chol or DPPC:DODAP:chol were found to be more efficient in contrast to those uncharged liposomes using DPPC only. The results showed that cholesterol played a key role in the interaction between DiMC and lipid bilayers by affecting their organization and consequently their stability, and incorporating DiMC into cationic liposomes was favored due to their thermodynamically stable liposomal dispersion. Moreover, the DPPC:DODAP:chol:DiMC liposomal formulation (9:1:1:1 molar ratio) was the most effective with an incorporation efficiency up to 3.84%, which reacted as an electrophile *via* the positive charge of the DODAP lipid molecule with ASC-J9 at phosphate buffer saline (pH 7.4).

Particle size effect on the anticancer ability of liposomes encapsulating DiMC also attracted research interest. The liposomes with different sizes were prepared by using the film dispersion technique, and then through the liposome extruder and dialysis. The results showed that the anticancer ability could be enhanced with the decrease in particle size, and the liposomes with a size of 184 nm could more efficiently induce the prostate cancer cell death than those with a size of 250.5 nm (Zhou et al., 2016).

Thereafter, various studies were performed on the preparation and properties of DiMC (namely, ASC-J9) liposomes (Li et al., 2018a; Li et al., 2018b; Sun et al., 2020). By using the thin-film dispersion method and using ethanol as a solvent, the optimal preparation conditions were obtained as the mass ratio of phospholipid to cholesterol of 10:1 and the mass ratio of phospholipid to DiMC of 1,000:10, with the hydration temperature set at 60 °C and the concentration of phospholipid at 6.67 mg/ml (Li et al., 2018a). This manufacturing technique had various advantages such as environmental friendliness, simple operation, and good product performance. In more detail, the liposomes can protect the entrapped DiMC well and be well dispersed in water and remain stable at 4 °C with the entrapment efficiency up to 97% and a mean particle size of 145 nm, with a very narrow particle size distribution (a polymer dispersity index of 0.36). The liposomes could be used as a water-soluble molecular fluorescence probe for the recognition of Fe^3+^, Fe^2+^, and Cu^2+^ through fluorescence quenching (Li et al., 2018b), as well as an inhibitor against cell proliferation *in vivo* and *in vitro* (Sun et al., 2020).

### Others

Inclusion complexes and solid dispersions are also involved in formulations of DiMC, mainly to improve its solubility and stability. By complexation with hydroxypropyl-γ-cyclodextrin, the commercial curcumin that contains DiMC could acquire increased solubility and stability (Benediktsdottir et al., 2015). Recently, the solid dispersions (SDs) of DiMC were prepared with polyethylene glycol (PEG) 4,000, PEG 6,000, and poloxamer 188 as carriers using the fusion method and with polyvinylpyrrolidone (PVP K30) as a carrier using the solvent evaporation method, respectively (Xu et al., 2019). The results showed that the drug dissolution rate could be significantly improved by all SDs, and the formulation using PVP K30 at a ratio to DiMC of 10:1 was the best one, where DiMC dispersed in an amorphous form with a cumulative dissolution of more than 83% in 5 min.

A kind of polymeric micellar formulation of DiMC for injection use in cancer therapy was first developed on the basis of the amphiphilic block copolymer with fairly low critical micelle concentration and passive targeting potential to tumor tissue (Liu et al., 2015). By using the copolymer mPEG-PCL-Phe (Boc), N-t-butoxycarbonyl-phenylalanine terminated monomethoxyl poly (ethylene glycol)-b-poly (ε-caprolactone) and the DiMC-loaded micelles could be easily prepared *via* the thin-film hydration method. Through high-affinity interaction between DiMC and the copolymer, the micelles had a typical shell-core structure with an average particle size of 17.9 ± 0.4 nm and a polydispersity index of 0.045 ± 0.011. The drug-loading capacity and entrapment efficiency were 9.94 ± 0.15% and 97.22 ± 0.18%, respectively. At a concentration of 2 mg/ml, the reconstituted micelle solution could be maintained for at least 10 days at room temperature and displayed a low initial burst release, followed by a sustained release *in vitro*. Pharmacokinetic study in rats revealed that *in vivo* drug exposure was significantly increased and prolonged by intravenously administering DMC-loaded micelles. This micellar formulation also greatly improved the biodistribution profile of DiMC and increased drug accumulation in tumors.

DiMC niosomes were recently prepared using the thin-film dispersion ultrasonic method, and the prescription composition and preparation process were optimized using the single-factor investigation method (Li et al., 2017c). The highest encapsulation rate was 88.1 ± 1.7%, and the drug-loading amount was 4.03 ± 1.05%. Moreover, the average particle size was 310.3 ± 0.9 nm, and the leakage rate was below 2% within 45 days, indicating that the niosomes as a vector could significantly improve the solubility and stability of DiMC.

Lately, novel stiffness-tunable nanocarriers were developed for the controlled delivery of the hydrophobic anticancer agent ASC-J9 into colorectal cancer cells (Tomeh et al., 2021). The core-shell nanocarriers composed of naturally derived polymers silk fibroin (SF) and sodium alginate (SA) inside a liposomal shell were prepared using the thin-film hydration method, followed by extrusion and cross-linking of SA to induce structural transformation of SF to obtain a uniform size and shape, avoiding harsh processing conditions. The nanocarriers had high encapsulation efficiency (62–78%) and were physically stable for up to 5 months at 4°C. The stiffness of the nanocarriers has a significant effect on drug release, cellular uptake, and anticancer efficacy. The release profile was directed by their stiffness and was easily tunable by changing the ratio of SF to SA in the core. Also, the designed nanocarriers improved the cellular uptake and anticancer activity of ASC-J9 and enhanced its tumor penetration in HCT 116 3D colorectal cancer spheroids, which thus can be used as a highly efficient drug delivery system for cancer therapy.

## Discussion and Conclusion

Natural products have been the most productive source for modern drug discovery and development. Curcumin is a kind of herb-derived natural polyphenol with various bioactivities such as anticancer activity. However, chemical modification of curcumin is usually needed since its systemic bioavailability and therapeutic potency are seriously constricted by poor solubility, low absorption, and hasty metabolism and elimination. DiMC is a striking curcuminoid derivative with significantly improved drug likeness and enhanced power. In the present review, we just systematically summarized the published research about DiMC as a promising anticancer agent, for which pharmacokinetics, pharmacological effects, molecular mechanisms, and nanoformulations for drug delivery were involved.

It has been demonstrated that the methylation of both free hydroxyl groups in curcumin provides the derivate DiMC with greatly improved biochemical stability and pharmacokinetic characteristics when compared with the parent molecule curcumin and other curcuminoids. Meanwhile, research has proven DiMC as a multi-target anticancer agent, which plays an exceptional role in the treatment of various malignant cancers through its superior effectiveness against cancer by triggering cell cycle arrest and apoptosis. More to the point, its efficacies toward cancer cell survival, attachment, movement, and invasion are closely related with the regulation of signal factors such as ROS, Cyt c, caspase-3/7/9, Bax/BCl2, GSH/GSSG, p53, and p21. It also has special chemosensitizing activity and photosensitizing property and thus could be developed as an anticancer adjuvant for cancer chemotherapy.

A reliable delivery system is usually one of the major challenges for drug development, and it is indeed the case for the anticancer agent DiMC. Despite the research on the subject, the drug delivery system of DiMC is still in its infancy; so far, a few nanoformulations have been investigated. Due to the great improvement in hydrophobicity, DiMC could be well incorporated by lipophilic substances to form liposomes, nanoparticles, and micelles, among which the polymeric micellar system is very promising as it can be easily produced with high drug-loading capacity, sustainable drug-releasing features, and satisfactory biodistribution. Recently, the development of complex nanoformulations dually-loaded adjuvant agent and classic first-line chemotherapeutic drugs such as doxorubicin and paclitaxel have attracted more and more research attention. Thus, we have no reason to doubt that such a novel drug delivery system of DiMC would provide an effective alternative to improving traditional cancer chemotherapies by acquiring synergic efficacy and overcoming adverse effects, especially some dose-limiting and cumulative toxicities, including doxorubicin-induced severe cardiac dysfunction.

In short, it is the first time that the basic information about research on DiMC has been comprehensively reviewed, and it will benefit in-depth development of this promising anticancer agent for clinical application in the near future.
